# Effect of caregiver burden on the quality of life of informal caregivers of people with cystic fibrosis in the United Kingdom: a cross-sectional study

**DOI:** 10.1007/s11136-025-04021-x

**Published:** 2025-07-18

**Authors:** Sulayman Chowdhury, Patricia Cubi-Molla, David Mott

**Affiliations:** Office of Health Economics (OHE), 2nd Floor Goldings House, Hay’s Galleria, 2 Hay’s Lane, London, SE1 2HB UK

**Keywords:** Quality of Life, Informal Caregivers, Cystic Fibrosis, Patient Reported Outcome Measures, Caregiver Burden

## Abstract

**Purpose:**

Informal carers of people with cystic fibrosis (PwCF) play a critical role in care provision, yet the impact of caregiving on their quality of life (QoL) remains underexplored. We aimed to assess the effect of caregiver burden on the quality of life of informal caregivers of people with cystic fibrosis in the UK.

**Methods:**

We conducted a cross-sectional online survey study administering a structured questionnaire with four validated measures (EQ-5D-5L, CarerQol-7D, ReQoL-10 and ASCOT-Carer). We used a carer-reported severity scale of cystic fibrosis to define severity groups. Statistical methods included descriptive analyses and ordinary least squares (OLS) regression to examine the association between carer utility and CF severity.

**Results:**

We find significant decrements in carers’ quality of life due to their care burden, with the most affected dimensions being mental health (79% of carers reported some anxiety or depression) and social health (60% reported negative impacts on social contact). We find this QoL to be significantly worse for those caring for people with severe CF compared to those with mild CF (−0.03 to −0.1), for the majority of the measures used (EQ-5D, ReQoL-10 and CarerQol-7D).

**Conclusion:**

Our paper shows the negative impact on QoL for carers of PwCF, correlated with increasing CF severity due to their carer duties, and the negative impacts on their various health aspects, especially mental health. This indicates the importance of including carer QoL and additional measures to fully capture burden in health technology assessments (HTA) for CF.

**Supplementary Information:**

The online version contains supplementary material available at 10.1007/s11136-025-04021-x.

## Introduction

Cystic fibrosis (CF) is a debilitating genetic condition currently affecting over 10,900 people in the United Kingdom (UK) [1]. Informal carers (those that provide unpaid care), often parents, play a crucial role in managing the care of people with CF (PwCF). This involves, but is not limited to, administering physiotherapy, medical treatments, and providing emotional and physical support [2]. More recent advances in CF treatment, such as CF transmembrane conductance regulator (CFTR) modulators, have improved patient survival, leading to an increasing proportion of adults with CF [3]. This shift impacts informal carers, traditionally parents, who may continue caregiving roles well into adulthood.

A systematic review of studies exploring carer burden in the context of CF [2] found that carer duties for PwCF led to a significant care burden across many countries, including the UK. This level of burden has wide-ranging impacts on carer wellbeing: leading to reduced sleep quality and reduced ability to work [4] and a reduction in health-related quality of life (HRQoL) [5], signifying a meaningful health loss for the carers. Furthermore, informal carers are more likely to have anxiety and depression than the general population [6], which tends to be correlated with the severity of the CF and adverse events such as hospitalisations. Addressing caregiver burden aligns with public health priorities such as the UK government plan for supporting carers [7] and the NHS long-term plan including improving mental health services [8].

This study aimed to add to the literature on carer burden in CF by examining the specific aspects of quality of life (QoL) that are impacted amongst a sample of informal carers in the UK. Quality of life (QoL) is a broad concept encompassing an individual’s physical health, mental well-being, social functioning, and economic stability [9]. Caregiver burden refers to the physical, emotional, and financial strain experienced by individuals providing unpaid care to a loved one [10]. To explore the QoL impacts of the provision of informal care in this context, multiple English language preference-based measures (PBMs) with established psychometric validity were used: EuroQol 5-Dimensions 5-Levels (EQ-5D-5L) [11], Recovering Quality of Life 10-item (ReQoL-10) [12], Care-related Quality of Life 7-Dimensions (CarerQol-7D) [13] and Adult Social Care Outcomes Toolkit for Carers **(**ASCOT-Carer) [14]. With this broad collection of measures, comprising of ‘generic’ measures (EQ-5D-5L) including a mental-health focused measure (ReQoL-10) alongside carer-specific measures (CarerQol-7D and ASCOT-Carer), we aimed to capture multiple aspects of carer health and QoL.

Alongside the broader examination of the QoL of carers of PwCF, a secondary aim was to explore how their QoL impact may differ depending on CF severity. Caring for individuals with more advanced CF may involve greater time demands, physical burden, and complex care tasks, potentially exacerbating impacts on carer wellbeing. Beyond these practical demands, carers may also experience psychological strain related to the distress and unpredictability of disease progression and concerns about future deterioration which may be worse with advanced stages of CF. This analysis also permits an exploration of the known group validity—a type of construct validity that tests differences between two or more groups with expected differences, for example, by levels of severity of the different measures and therefore enables some consideration of their relative suitability in this context. There is no single universal measure for categorising CF severity with different scoring systems [15, 16], PROMs or medical factors such as Forced Expiratory Volume as a % of predicted normal FEV (FEV%) being used instead. We explore the use of a carer-reported severity scale for this purpose. With this scale and the selected outcome measures, we assess the effect of caregiver burden on the quality of life of informal caregivers of people with cystic fibrosis in the UK.

## Methods

### Survey

This study used an analytical cross-sectional design through an online survey administered to informal carers of PwCF (see Appendix 1) between July–August 2020. The survey began with general questions about the carer. These questions included their relation to the PwCF, some clinical characteristics of the PwCF (including comorbidities and disease severity), their caring responsibilities (including time spent providing care), and demographics questions. The carer was then asked to assess their health using four validated PBMs: EQ-5D, ReQoL-10, CarerQol-7D and ASCOT-Carer. The order in which the PBMs were shown was randomised to mitigate the effects of fatigue on responses to each measure and to minimise biases due to question order effects [17]. Participants were recruited using convenience sampling through CF Voices, an organisation led by carers of PwCF in the UK. Eligibility criteria required participants to be adults who were actively providing care for a PwCF at the time of the study, a criterion inherently met by all members of the organisation. Ethical approval for the study was provided by the Senate Research Ethics Committee at City, University of London (reference: ETH-1920-1907).

### Validated instruments

#### EQ-5D-5L

Developed by the EuroQol Group [18], the EQ-5D-5L is a ‘generic’ measure that consists of a questionnaire covering five dimensions: mobility, self-care, usual activities, pain/discomfort, and anxiety/depression, each with five response levels from ‘no problems’ to ‘extreme problems’. The second part is a visual analogue scale for self-assessing health. The EQ-5D-5L instrument is intended to gauge *health-related* QoL, potentially neglecting other aspects of QoL like feelings of safety, happiness, and caregiving experiences. However, it was incorporated into the survey because it is widely regarded as the foremost patient-reported outcome measure for health technology assessments globally [19] and is therefore commonly used to inform reimbursement decisions. Notably, given our study population, the National Institute for Health and Care Excellence (NICE) recommend that EQ-5D is used to capture impacts on the health of carers as well as patients [20].

#### ReQoL-10

Given that carer burden is likely to have a greater impact on mental health than physical health [21–23], we also included ReQoL-10;a ten-item generic mental health PBM [12] that asks respondents about their experiences over the previous week. The dimensions include: difficulty getting started, trust, coping, ability to do own activities, happiness, self-esteem, enjoyment, hope, loneliness, and confidence. The dimensions are phrased in the form of positive/negative questions, each dimension has five response levels ranging from “none of the time” to “most or all of the time” as the strongest response, thus a strong response can either be a positive or negative implication depending on the phrasing. This measure was included in the survey because of its focus on mental health, which may be more relevant for carers than physical health, the latter of which is a greater focus in the EQ-5D-5L. It includes more granular elements of mental health compared to EQ-5D and could therefore provide further insights into the various aspects of mental health QoL for carers.

#### CarerQol-7D

Alongside these generic measures, we also included carer-specific PBMs that assess carer burden directly. CarerQol-7D is a carer-specific measure developed for assessing carer’s QoL [13] across seven dimensions surrounding their caregiving situation: fulfilment, relational problems with the patient, mental health, daily activities, financial problems, support with their care duties, and physical health. This measure was included in the survey as it is a validated measure [24] that focuses specifically on the burden of care and has been used before for carers of PwCF[2], allowing insight into the impact of specific aspects of their care burden.

#### ASCOT-Carer

ASCOT-Carer measures the social care-related QoL of informal carers across seven dimensions: time for leisure, control, self-care, safety, social contact, personal space/time, and support. Each dimension has four response levels, that ranges from an ideal situation to a severe negative situation. ASCOT-Carer, while gaining popularity as a tool dedicated to addressing social challenges encountered by informal carers, has seen limited application in the context of CF, though is validated in other disease areas [25]. Evidence from other disease domains indicates its potential to outperform CarerQol-7D [26, 27].

### Statistical analysis

To compare the distribution of the responses to the four measures, the responses were converted to utilities using value sets; utility values represent quality of life on a continuous scale from 0 (worst state) to 1 (best state) with different measures measuring different aspects of quality of life. Specifically, the EQ-5D-5L crosswalk value set developed by van Hout et al. [28], the ReQoL-10 value set developed by Keetharuth et al. [29], the CarerQol value set developed by Hoefman et al. [30], and the ASCOT-Carer value set developed by Batchelder et al. [31]. The utilities were analysed using Stata version 13.1® [32] through general mean and distribution analysis and ANOVA.

Severity of the CF experienced by the PwCF was reported by carers as either mild, moderate, or severe. Given the subjective nature of the scale, we sought to validate its use by comparing assessments based on more objective indicators that signal CF severity, such as the % Forced Expiratory Volume (%FEV1) which previous studies have shown to decrease as CF severity increases [33, 34]. Furthermore, we explored whether factors that in theory should not be related to the severity of CF were nevertheless influencing our severity variable, to further test its validity.

1 $${Y}_{i}={\alpha }_{i}+{\beta }_{1}Severity+{\varepsilon }_{i}$$

where ${Y}_{i}$ represents the objective factor being explored and $Severity$ is a categorical variable equal to 1 for mild, 2 for moderate, and 3 for severe CF. A significant beta coefficient would indicate that there is a relationship between the objective factor and carer-reported severity.

We explored how QoL in the carer differed by CF severity by conducting multivariate ordinary least squares (OLS) regression analyses. We controlled for additional factors, such as the number of other carers (apart from the primary carer filling out the form) and the daily overall care hours spent by the carer (as opposed to daily hours of medical-related care which would likely result in issues with collinearity with the severity scale). Demographic factors such as sex and education were shown to be correlated with the severity scale and were thus excluded to avoid collinearity.

We estimated a regression model for each QoL measure, as set out in Eq. 2:

2 $$QOL_{i} = \alpha_{i} + \beta_{1} Severity_{Moderate} + \beta_{2} Severity_{Severe} + \beta_{3} Daily\;Care\;Hours + \beta_{4} No.\;of\;Other\;Carers + \varepsilon_{i}$$

where ${QoL}_{i}$ represents the QoL scores, ${\alpha }_{i}$ is a constant term, $Severity$ is a categorical variable equal to 1 for mild, 2 for moderate, and 3 for severe CF, *Daily Care Hours* is a continuous variable representing the number of daily care hours provided by the carer, and *No. of Other Carers* is the number of other individuals that provide regular informal care to the PwCF (self-reported). The severity variable is split into two dummy variables, indicating whether the carer reported that the PwCF has moderate or severe CF. Thus, this model explores the linearity of the QoL decrements between the severity levels by indicating how QoL scores decrease on average between those self-reporting moderate CF (compared to mild CF) and between those self-reporting severe CF (compared to mild CF).

## Results

A total of 258 informal carers of PwCF from the UK were recruited for the study. The demographic information of the carers and the characteristics of those that they provide care for are detailed in Table 1. The majority of carers were female (n = 219; 85%), described themselves as the ‘primary’ carer (n = 229; 89%), were a parent of the PwCF (n = 240; 93%), and had some form of higher education (n = 213; 83%). On average, most carers reported having one other carer for the PwCF. Around a fifth of carers (n = 50; 19%) reported providing care full-time, with just over half (n = 147; 57%) of the carers being in full or part-time work. The mean hours of paid work were 18.2 h weekly, alongside 10.4 h (on average) caring for the PwCF daily, of which 3.2 h were specifically for medical care. The demographics of the PwCF show an even split between males and females with the majority of carers reporting that the PwCF had ‘moderate’ severity (n = 147; 57%). On average, PwCF suffered from around two other comorbidities alongside, or because of, CF; the most common being pancreatic insufficiency (n = 189; 73%) and anxiety/depression (n = 85; 33%).

**Table 1:** Carer and PwCF demographics

Parameter	Frequency	% of Sample
	(n = 258)	(rounded)
**Carer demographics**		
** Sex**		
Male	31	12%
Female	219	85%
Prefer not to say	8	3%
** CF in carer**		
Yes	0	0%
No	258	100%
** Employment**		
Full-time worker	72	28%
Part-time worker	74	29%
Full-time carer (informal)	50	19%
Homemaker	28	11%
Student	3	1%
Self-employed	17	7%
Retired	5	2%
Other	9	3%
**House income**		
Under £20,000	64	25%
£20,000–£50,000	113	44%
Over £50,000	65	25%
Prefer not to say	16	6%
**Education**		
No higher education (A-Level or equivalent)	45	17%
Higher education (A-Level or equivalent)	213	83%
No degree or equivalent	111	43%
Degree or equivalent	147	57%
**Primary carer**		
Yes	229	89%
No	27	10%
Not sure	2	1%
**Relation to PwCF**		
Parent	240	93%
Spouse	9	3%
Sibling	1	0.40%
Friend	1	0.40%
Other family	4	2%
Other	3	1%
**Mean hours providing care (per day)**	10.4 (Range: 0–24)	
Mean hours specifically for medical treatment	3.2 (Range: 0–18)	
**Mean no. other carers to PwCF**	1 (Range: 0–4)	
**Mean hours of paid work (per week)**	18.2 (Range: 0–60)	
**PwCF demographics**		
**Sex**		
Male	121	47%
Female	124	48%
Prefer not to say	13	5%
** CF severity**		
Mild	75	29%
Moderate	147	57%
Severe	36	14%
**CF gene modulator treatment**		
Yes	146	57%
Starting in the next two months	18	7%
Starting in the next two months	51	20%
Genotype not responsive to modulator	18	7%
I don’t know/Prefer not to say	25	10%
**CF mutation combination**		
F508del + F508del	152	59%
F508del + Other	91	35%
Other + Other	11	4%
Don’t know	4	2%
**Mean FEV1 %**	77.2	
**Mean no. comorbidities due to CF**	2.4 (Range: 0–8)	
** Comorbidity frequency**		
Pancreatitis	8	3%
Pancreatic insufficiency	189	73%
Gastro-oesophageal reflux disease	60	23%
Male infertility	30	12%
Urinary stress incontinence	15	6%
Rhinosinusitis and or nasal polyps	66	26%
Gallbladder anomalies/disease	9	3%
(CF-related) liver disease	45	17%
(CF-related) diabetes	44	17%
Anxiety/depression	85	33%
Osteopenia/osteoporosis	28	11%
Other	38	15%
**Mean no. hospital admissions (last year)**	1.3 (Range: 0–12)	
Mean no. unplanned hospital admissions	0.7 (Range: 0–9)	

*CF* cystic fibrosis, *PwCF* person with cystic fibrosis, *No.* number of, *FEV1* forced expiratory volume in 1 s

**Fig. 1:**
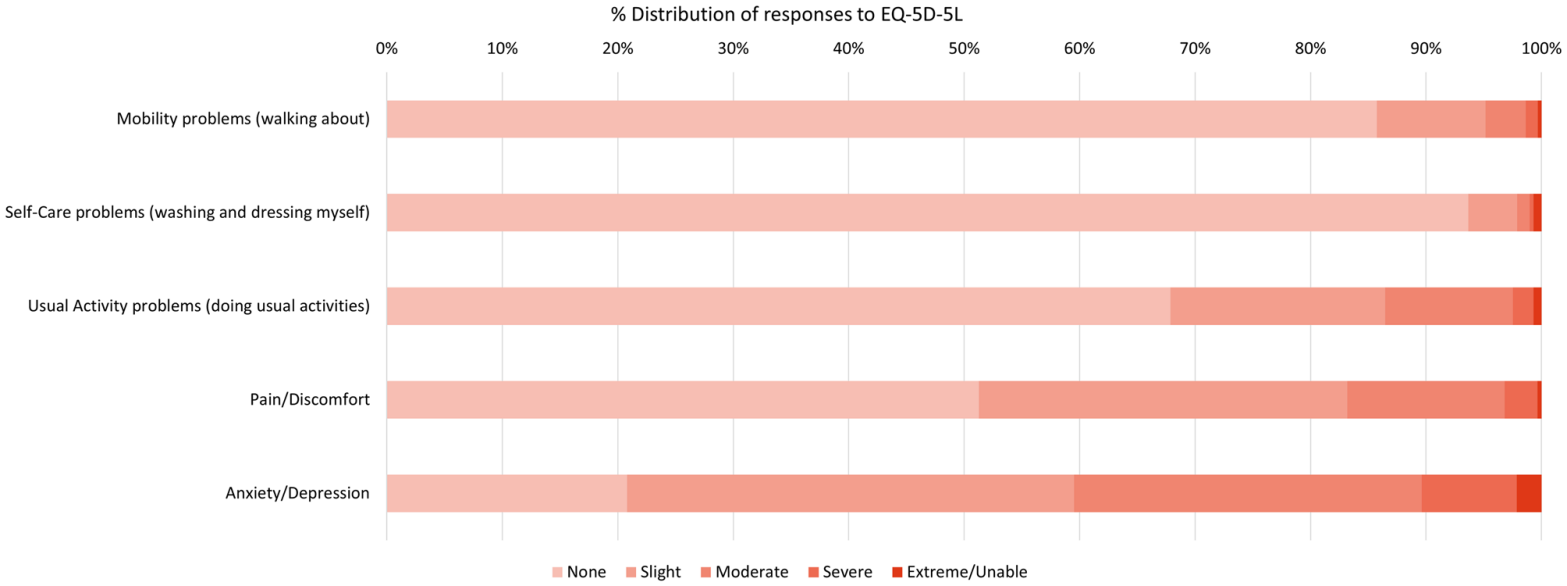
% Distribution of responses to EQ-5D-5L dimensions

**Fig. 2:**
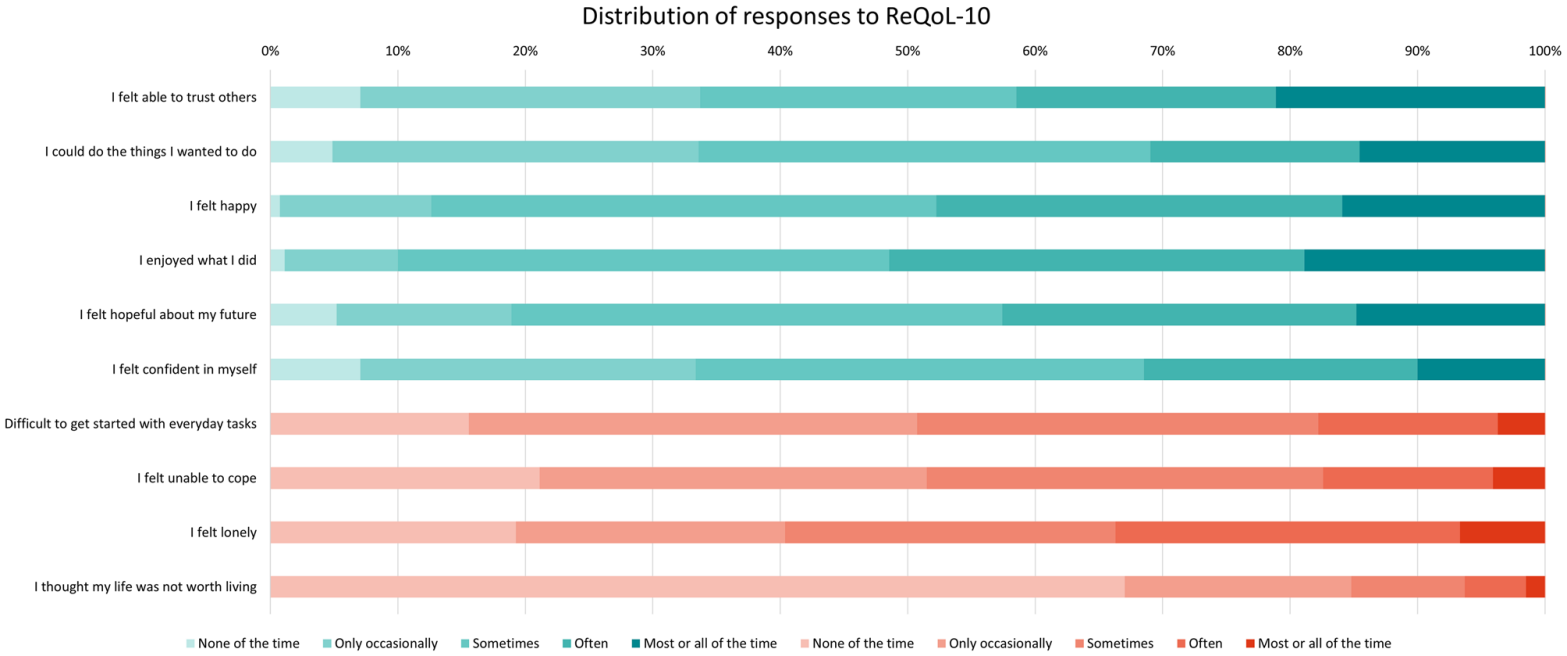
% Distribution of responses to ReQoL-10 dimensions with colour coding for positively-framed dimensions and negatively-framed dimensions

**Fig. 3:**
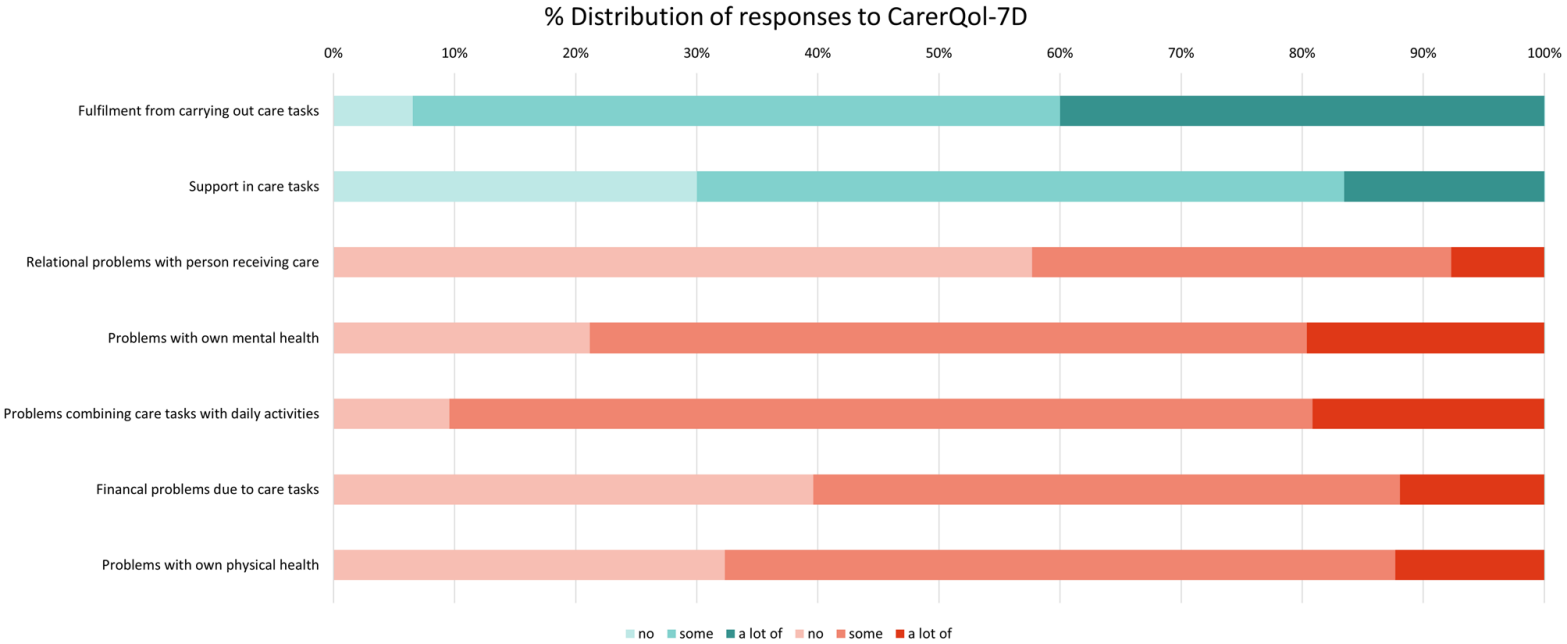
Distribution of responses to CarerQol-7D dimensions

**Fig. 4:**
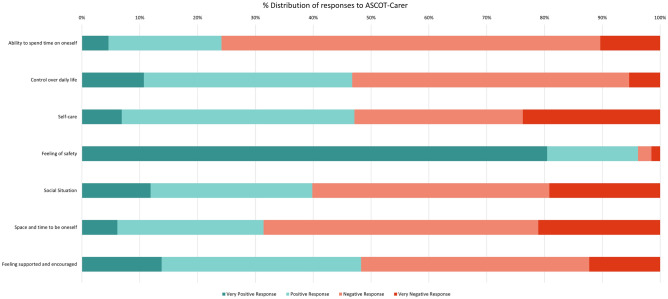
% Distribution of responses to ASCOT-Carer dimensions

The distribution of responses to all four measures is presented in Figs. 1, 2, 3, 4. Figure 1 provides the EQ-5D-5L responses, which are based on the respondents’ health at the time of completion. Responses to EQ-5D-5L dimensions of mobility and self-care were largely positive with over 90% of carers reporting no or slight problems. Responses to problems with usual activities show a larger proportion of respondents reporting slight to severe problems (30% of carers). Nearly half of the carers reported slight to severe problems with pain/discomfort, the majority of which were reporting slight problems. The majority of carers, around 79%, reported problems with anxiety/depression with approximately 40% having moderate-severe responses and 2% responding at the extreme level.

For ReQoL-10, severity interpretation differs, and the recall period is one week. In Fig. 2, positive dimensions are blue, and negative ones are orange. The most reported issue was loneliness: approximately 60% felt lonely at least sometimes, approximately 25% often, and approximately 7% most or all of the time. Conversely, over half enjoyed their daily activities most or all the time. More than 30% struggled with trust, doing desired activities, and self-confidence. While two-thirds never felt life was not worth living, a third did at least occasionally, with a small proportion feeling this way often or most of the time.

CarerQol-7D also has positively and negatively-framed questions, and the respondents are asked to reflect on their caregiving situation in the moment. A large proportion of carers reported having some, or a lot of, ‘problems with their mental health’ (almost 80%) and ‘problems combining care tasks with daily activities’ (just over 90%). Furthermore, a significant proportion of respondents reported some, or a lot of, ‘problems with own physical health’ (around 67%), ‘financial problems due to care tasks’ (60%), and ‘relational problems with the person receiving care’ (just over 40%). In contrast, 40% of carers reported having a lot of ‘fulfilment from carrying out care tasks’ and more than 50% of carers reported having some fulfilment. Furthermore, 70% of carers reported having some or a lot of ‘support with carrying out their care tasks’.

ASCOT-Carer questions are all positively-framed. Responses to individual items are discretely worded and do not conform with each other so have been coded from “very positive” responses to “very negative” responses for the analysis. Around 75% of respondents gave a negative response regarding having time to do things they enjoy or value. Just over half of carers gave a negative response to having control over their daily life and to how well they look after themselves. Around 95% of carers gave a positive response to feeling safe. Approximately 60% gave negative responses regarding how much social contact they can have with others, around 70% reported feeling that they do not have enough or any time and space to be themselves, and around half of the respondents feel that they do not have adequate or any encouragement and support.

**Fig. 5:**
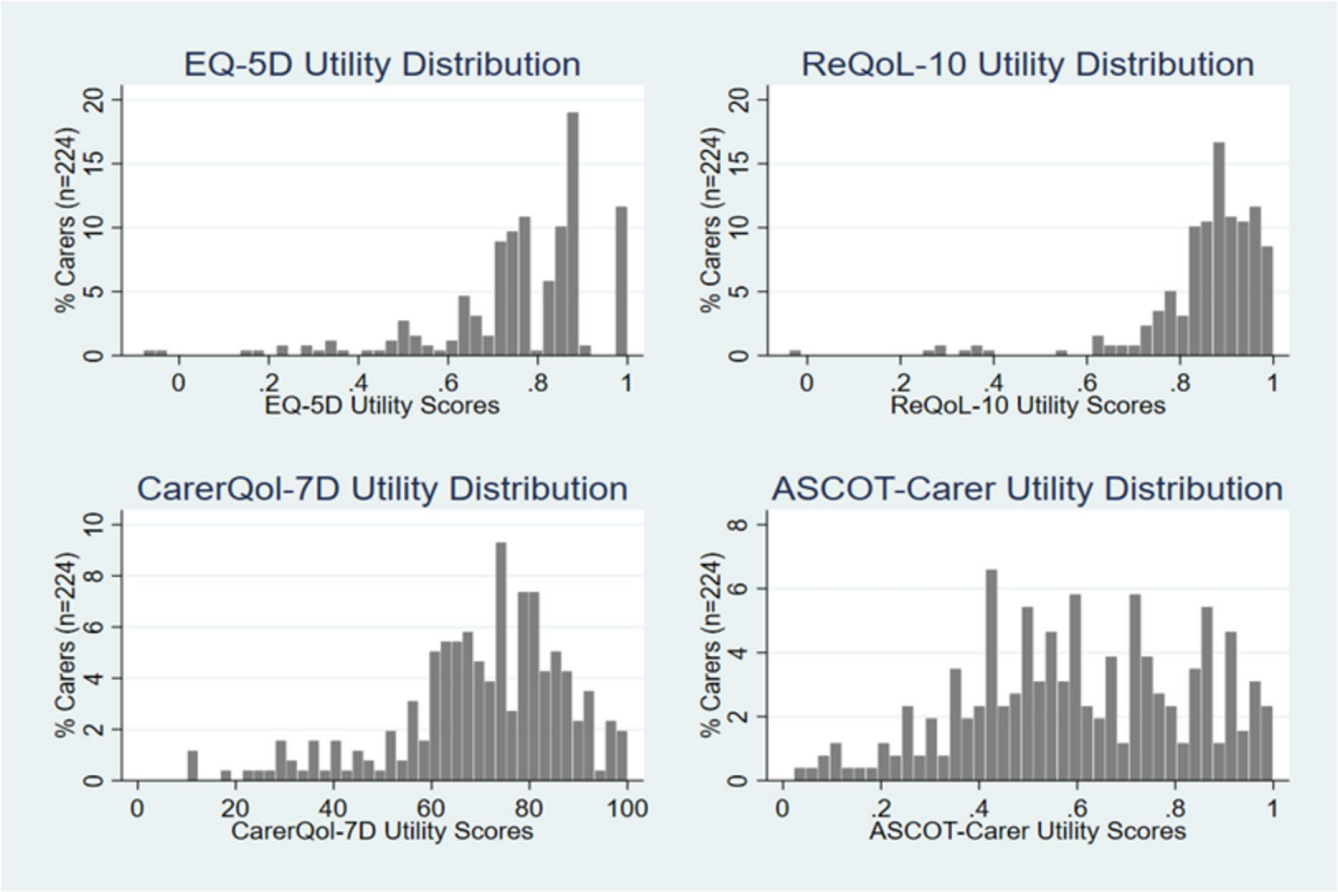
Distribution of utility scores across EQ-5D (Mean Score: 0.76), ReQoL-10 (Mean Score: 0.86), CarerQol-7D (Mean Score: 70), and ASCOT-Carer (Mean Score: 0.60)

The distributions of utilities for each instrument after applying the value sets are shown in Fig. 5. All instruments show largely positively-skewed distributions of utilities, with EQ-5D-5L (mean score: 0.76) and ReQoL-10 (mean score: 0.86) showing the strongest right skews followed by CarerQol-7D (mean score: 70). ASCOT-Carer (mean score: 0.60) has the widest spread of values, with significantly more values under 0.5 compared to the other instruments. Ceiling effects—where respondents report no problems/issues on any dimensions—were not common in this sample overall but were most common for EQ-5D-5L (around 11–12% responses) and ReQoL-10 (around 8–9%).

To validate carer-reported CF severity, we examined its sensitivity to expected and unexpected factors. Table 2 shows regression results, with severity increasing alongside worsening health indicators (%FEV1, comorbidities, medical care hours, work hours, and hospital admissions). As expected, the number of other carers and total care hours were not significant. These findings support using carer-reported severity to assess its impact on carer QoL.

**Table 2:** Regression results for assessing carer-reported severity

Dependent variable	Coeff	SE	p-value	95% CI	Model R^2^
No. of comorbidities in PwCF	1.13	0.15	0.000	[0.85; 1.42]	0.165
%FEV1 of PwCF^a^	−22.5	2.81	0.000	[−28.09; −16.98]	0.362
No. of other carers	0.20	0.38	0.593	[−0.55; 0.95]	0.003
Daily hours spent on care	−0.17	0.71	0.809	[−1.57; 1.23]	0.000
Daily hours spent on medical care	0.86	0.19	0.000	[0.49; 1.24]	0.048
Weekly hours of paid work	−5.81	1.62	0.000	[−9.00; −2.62]	0.046
No. of planned hospital admissions in last year	1.38	0.19	0.000	[0.99; 1.76]	0.226
No. of unplanned hospital admissions in last year	0.75	0.14	0.000	[0.46; 1.02]	0.133

*Coeff* coefficient on the severity scale variable, *SE* robust standard error, *CI* confidence interval

^a^%FEV1 was only provided by a subset of carers (n = 109)

OLS regressions reveal utility decrements across all four QoL instruments by carer-reported CF severity. Relative to those reporting mild CF severity, utilities on each measure were lower on average for those reporting moderate or severe CF severity, as indicated by the negative coefficients in all models reported in Table 3. The model results provide some insight into the linearity of the decrements. EQ-5D-5L and ReQoL-10 utilities were lower on average, and to a similar degree, for those reporting moderate or severe CF. In contrast, the utilities reported on average by those reporting moderate CF were not statistically significantly different from those reporting mild CF. However, the utilities were significantly lower on average for those reporting severe CF compared to those reporting mild CF. The auxiliary care burden factors (daily care hours and number of other carers) appear to also have a significant impact (albeit at varying significance levels) on the utilities for all instruments except EQ-5D-5L.

**Table 3:** Regression Results for independent variables against utility value, for each instrument

	EQ-5D-5L	ReQoL-10	CarerQol-7D	ASCOT-Carer
Moderate CF (Dummy)	−0.0388* (0.0233)	−0.0347** (0.0164)	−0.0348 (0.0221)	−0.0214 (0.0305)
Severe CF (Dummy)	−0.0744** (0.0414)	−0.0584** (0.0242)	−0.104** (0.0409)	−0.0910* (0.0464)
Daily care hours	−0.0025 (0.0016)	−0.0018 (0.0011)	−0.0039*** (0.0014)	−0.0049*** (0.0018)
No. of other carers	0.0025 (0.0031)	0.0041** (0.0020)	0.0113*** (0.0022)	0.0128*** (0.0026)
Constant	0.819*** (0.0241)	0.900*** (0.0147)	0.763*** (0.0235)	0.665*** (0.0291)
R^2^	0.030	0.037	0.077	0.055
N	258	258	258	258

Robust standard errors in parentheses

***p < 0.01, **p < 0.05, *p < 0.1

## Discussion

### QoL impacts

This study contributes to the literature on the QoL impacts of the provision of informal care for PwCF. We included various validated preference-based instruments to measure different aspects of carer QoL.

Overall, our results suggest that the provision of informal care for PwCF impacts carers’ QoL. These findings align with previous studies on informal caregiving in CF. For example, Daly et al. (2022) [2] found that informal carers of PwCF experience significant health-related burden, particularly in mental well-being. Similarly, Quittner et al. (2014) [6] highlighted that parents of PwCF have elevated levels of anxiety and depression, supporting our findings on mental health impact. More broadly, Our results also complement broader research for other diseases, such as Lacey et al. (2024) [21], which demonstrated that caregiving is associated with worsening physical and mental health outcomes over time. Likewise, Chang et al. (2010) [23] reported that caregiving for patients with dementia led to increased anxiety and physical health deterioration, suggesting a broader trend across caregiving contexts. The effects of caregiver burden on carer QoL has been shown to be significant in many other disease areas recently [35, 36].

The EQ-5D-5L results suggest the most impacted dimension is anxiety/depression. The ReQoL-10 results provide further insight into the mental health of carers, suggesting that many feel lonely, constrained in their ability to do the things that they want to do, as well as struggling to trust others and feel confident in themselves. A small but significant proportion of carers report occasionally thinking that their life is not worth living, signifying the severity of this mental impact. The carer-specific measures included in the study provide some indication around what may cause these mental health impacts. The CarerQol-7D responses illustrate that most carers have some, or a lot of, problems combining care tasks with their daily activities. Many also report financial problems due to care tasks, and very few respondents felt that they are supported in their care tasks. These general insights are reiterated in the ASCOT-Carer responses. Most respondents are the primary carer, with the PwCF being their child. With the reported number of hours per day for providing care (> 10 on average), coupled with the severity of the CF (> 70% being moderate or severe) and the frequency of comorbidities in the PwCF, it is evident that this sample of carers experience a substantial caring burden. A smaller proportion of carers (7%) were caring for an adult with CF, either as a spouse, sibling, or other relative. These carers may experience different challenges, including navigating adult healthcare services and government processes on behalf of the PwCF, balancing employment with caregiving, and dealing with age-related comorbidities and disease progression [3, 21].

Nevertheless, the CarerQol-7D responses indicate strongly that the overwhelming majority of carers do get fulfilment from carrying out care tasks despite experiencing negative health impacts themselves.

### The impact of CF severity

We also explored the impact of disease severity on carer QoL. In CF, there is not a universally accepted severity scale. We opted to collect carer-reported data on CF severity as our primary measure of severity, using three categories: mild, moderate, and severe. To test the validity of this scale, we also collected data on a range of CF characteristics and explored how sensitive the scale was to these characteristics. We found the scale to be robust with significant correlations with measures of CF progression such as %FEV1 and previous hospital admissions. This suggests that carers of PwCF have an accurate insight into the severity of CF in the person that they care for.

Our results suggest that CF severity does have a significant negative impact on carer QoL according to nearly all measures, as might be expected. Although the utilities from each measure are not directly comparable, it is notable that the magnitude of the impact, which is relatively small, is similar for each measure. The level of sensitivity varied by measure. It appears that the impact of severity on the carer-specific measures is largely driven by those reporting severe CF, whereas in contrast, the generic measures appear to be more sensitive to the different levels of severity. A possible explanation is that the experience and provision of care is not substantially different for carers of people with mild or moderate CF, but that the mental health impact of caring for a PwCF with moderate CF is greater than with a PwCF with mild CF, perhaps due to the progressive nature of the disease. Alternatively, it may simply be the case that the carer-specific measures are less sensitive to milder care burdens, perhaps due to the wording of the dimensions and response levels, or perhaps even due to the lower number of response levels (five for EQ-5D-5L and ReQoL-10 vs. three and four for CarerQol-7D and ASCOT-Carer respectively).

### Strengths and limitations

This study has several strengths. Firstly, our sample size is large considering the relative rarity of the disease. Also, we have collected data using four validated measures that cover different constructs. This range of measures enabled us to obtain a broader picture of carer QoL, and the detrimental effects that caring duties can have on QoL. Furthermore, we intentionally focused on collecting data using preference-based measures, as these are more relevant for health technology assessment, and relatively few studies have used these measures in this context to date.

This study has several limitations. First, we used convenience sampling via CF Voices, a parent-centric organization, limiting generalisability to the broader CF carer population. Second, data were collected in July–August 2020, when COVID-19 restrictions were still in place. This may have exacerbated reporting of limited activities, loneliness, and mental health issues, potentially biasing results and making QoL appear worse than in a ‘normal’ period. Meaningful comparisons with ‘carer norms’ would require data from the same time. Additionally, carer age was not robustly collected, preventing age adjustment in our models. However, we have no reason to believe carer age varied significantly by CF severity, given the parent-focused recruitment.

### Future research

Further research is advised now that more time has passed since the UK’s COVID-19 lockdowns. We recommend using multiple measures to assess carer QoL, including EQ-5D-5L for general health assessments and ReQoL-10 for mental health. CarerQol-7D appears more sensitive to disease severity than ASCOT-Carer in our study, though both may be useful. The recently developed EQ-HWB [37] could also provide valuable insights. Longitudinal research would further clarify the impact of care provision and the performance of QoL measures especially with new CF treatments likely shifting the landscape of caregiving duties.

## Conclusion

Our findings reveal a substantial caring burden for those that care for PwCF. Using validated preference-based measures, we find that this burden takes a significant toll on the QoL of carers of PwCF, primarily concerning their mental health. The impact on QoL appears to be greater for those providing care for people with more severe CF, and this finding is consistent regardless of the measure used. This reinforces the importance of including carer QoL in HTA assessments for CF as well as using additional measures alongside the EQ-5D.

## Supplementary Information

Below is the link to the electronic supplementary material.

Supplementary file1 (DOCX 1189 KB)

## Data Availability

The datasets generated and analysed during the study are not publicly available in an open repository. However, anonymised data may be made available from the corresponding author on reasonable request, subject to approval by the study team and in accordance with participant consent and ethical approvals.
