# Supplementary material for: Effect of caregiver burden on the quality of life of informal caregivers of people with cystic fibrosis in the United Kingdom: a cross-sectional study
Source: Qual Life Res. 2025 Jul 18;34(10):2995–3006. doi: 10.1007/s11136-025-04021-x (PMC12535523; doi:10.1007/s11136-025-04021-x)

Journal Article Title: Assessing the impact of carer burden on the quality of life for carers of people with cystic fibrosis in the United Kingdom

Authors and Affiliations:

Sulayman Chowdhury^1^ (ORCID ID: 0000-0001-8468-2122),

Patricia Cubi-Molla^1^ (ORCID ID: 0000-0002-2803-7337),

David Mott^1^ (ORCID ID: 0000-0001-5959-8447)

Corresponding Author:

Sulayman Chowdhury^1^

[schowdhury@ohe.org](mailto:schowdhury@ohe.org); sulayman.c@hotmail.com

^1^ Office of Health Economics (OHE) | 2nd Floor Goldings House, Hay’s Galleria, 2 Hay’s Lane, London, SE1 2HB, United Kingdom.

Appendix 1 – Online Survey Screenshots


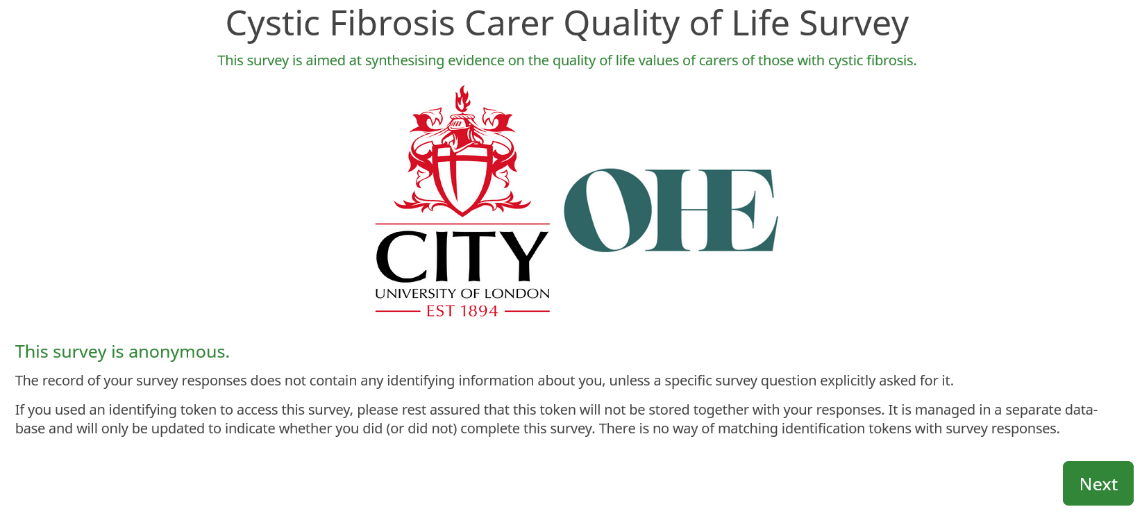


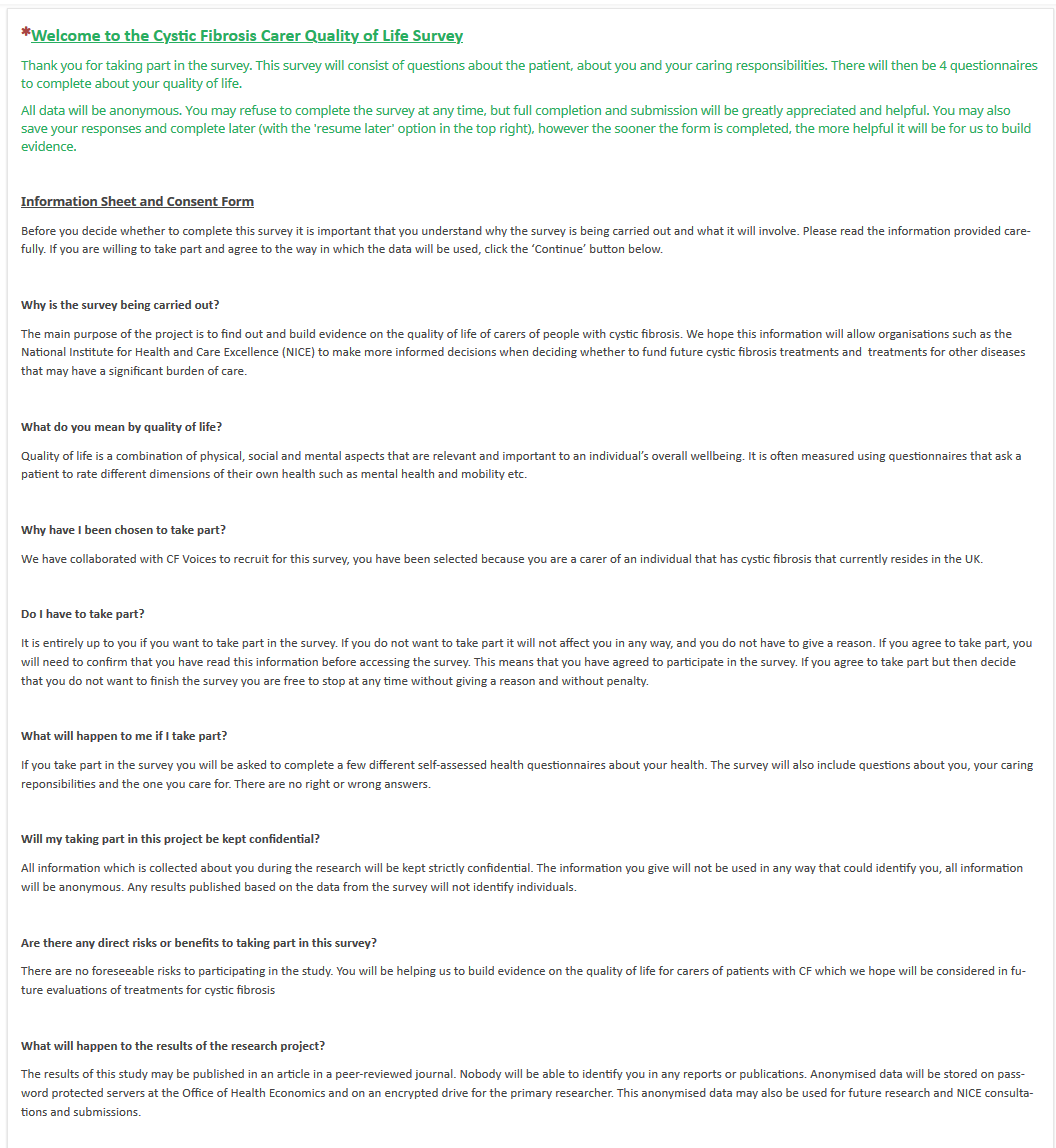


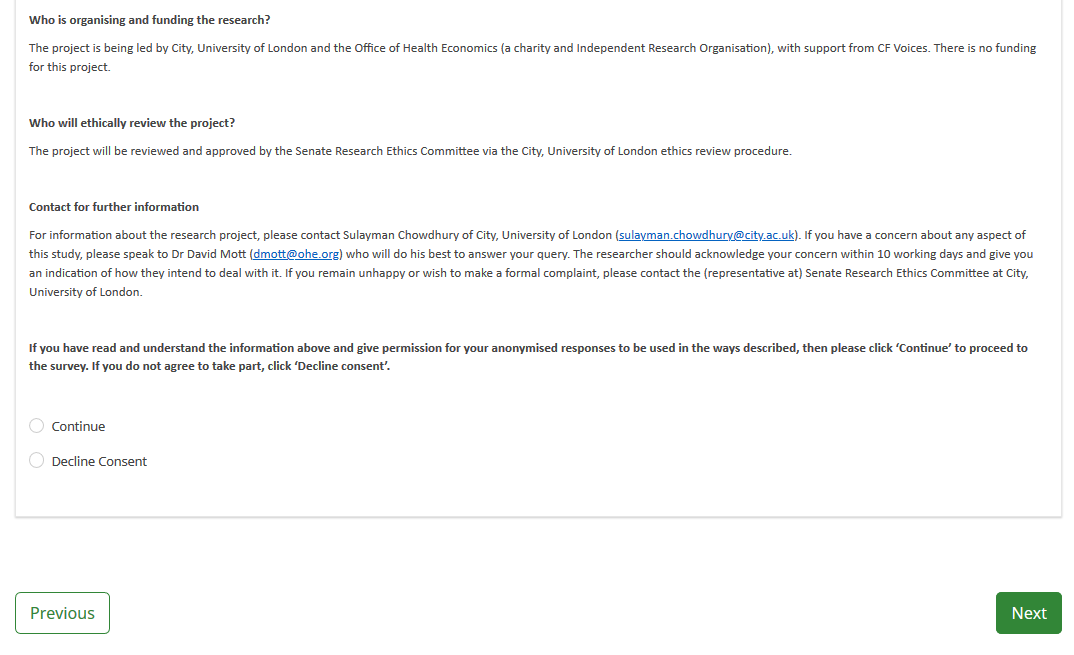


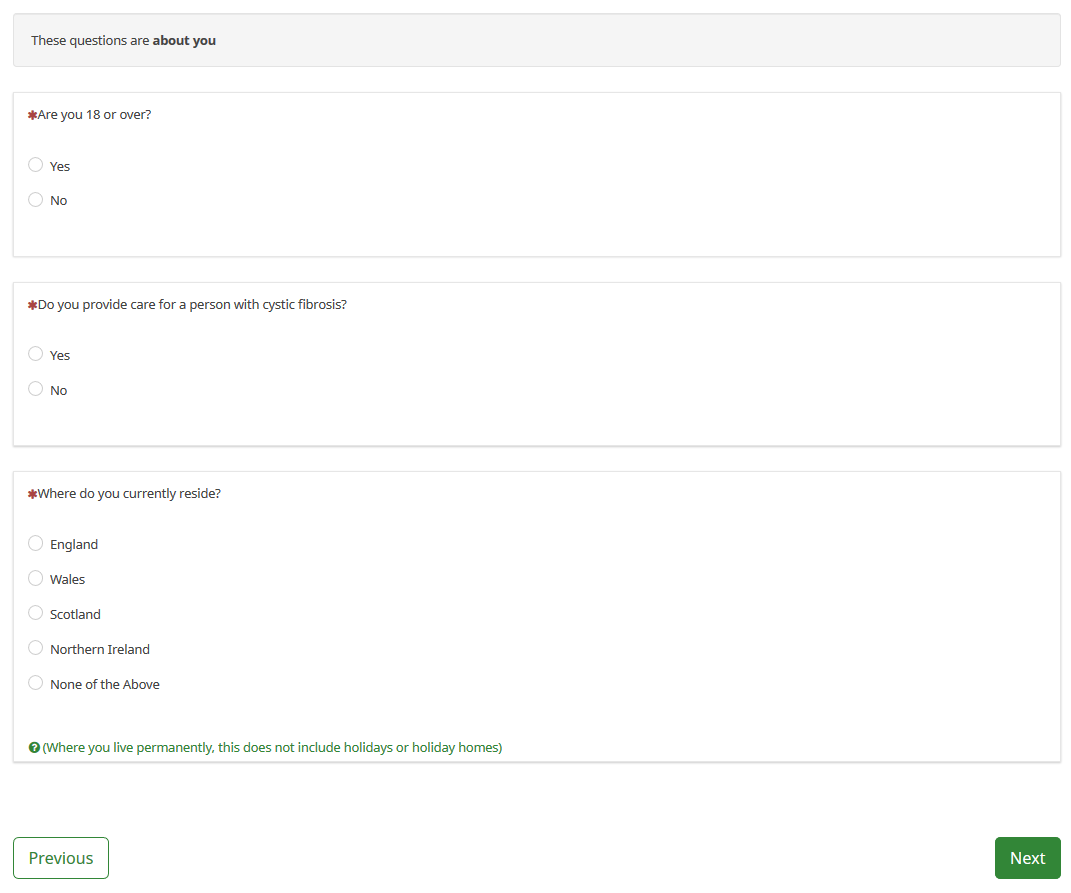


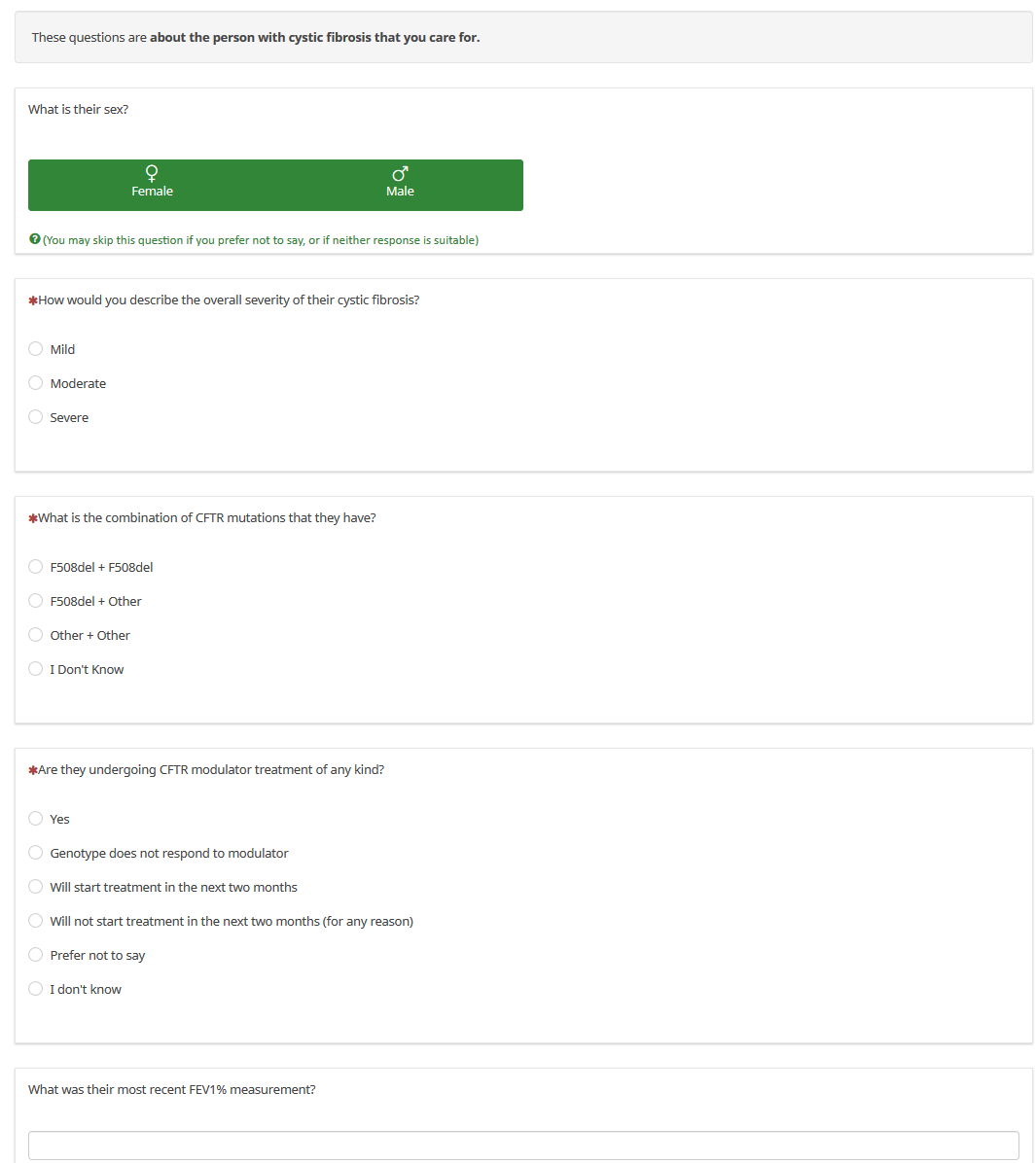


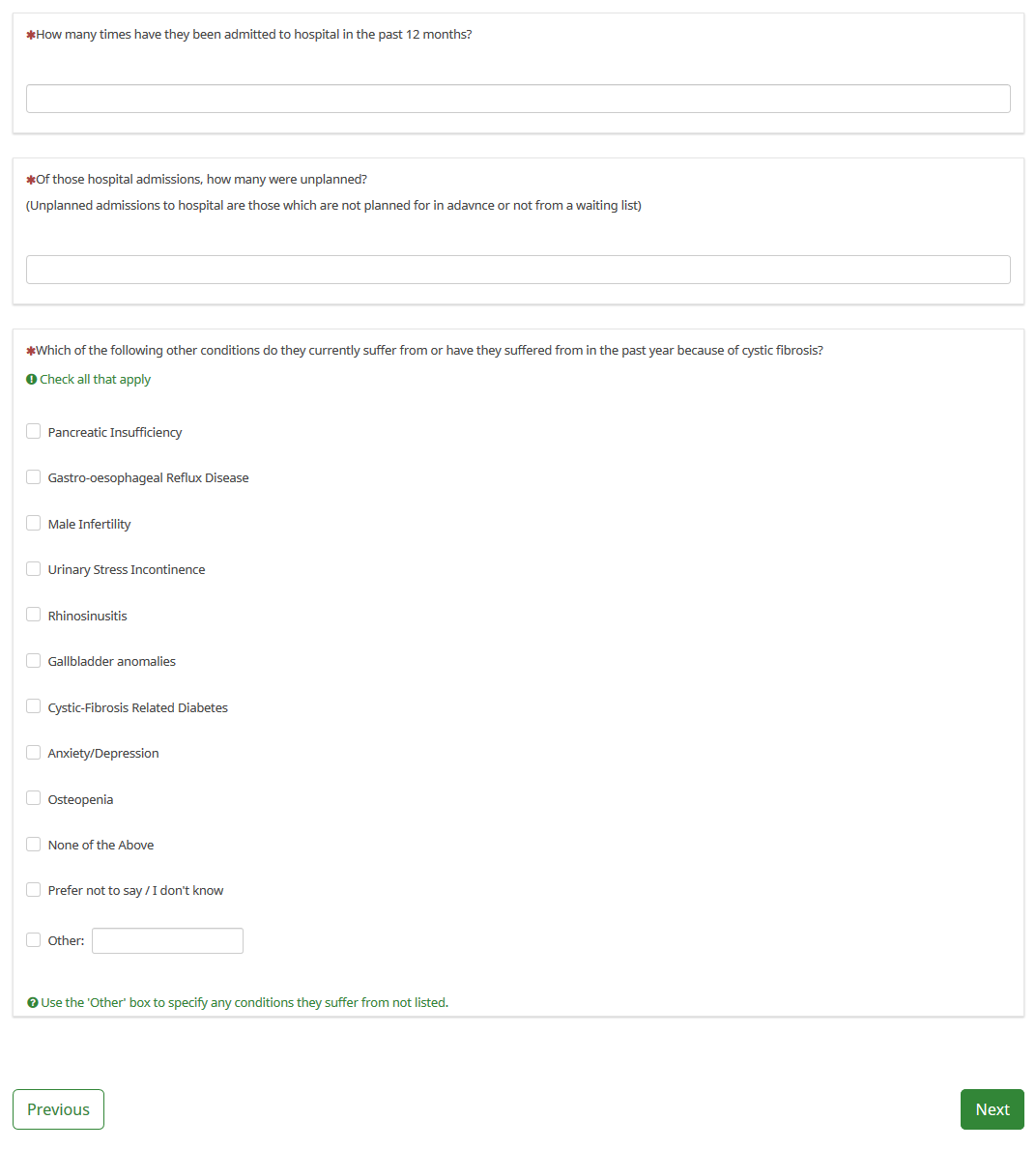


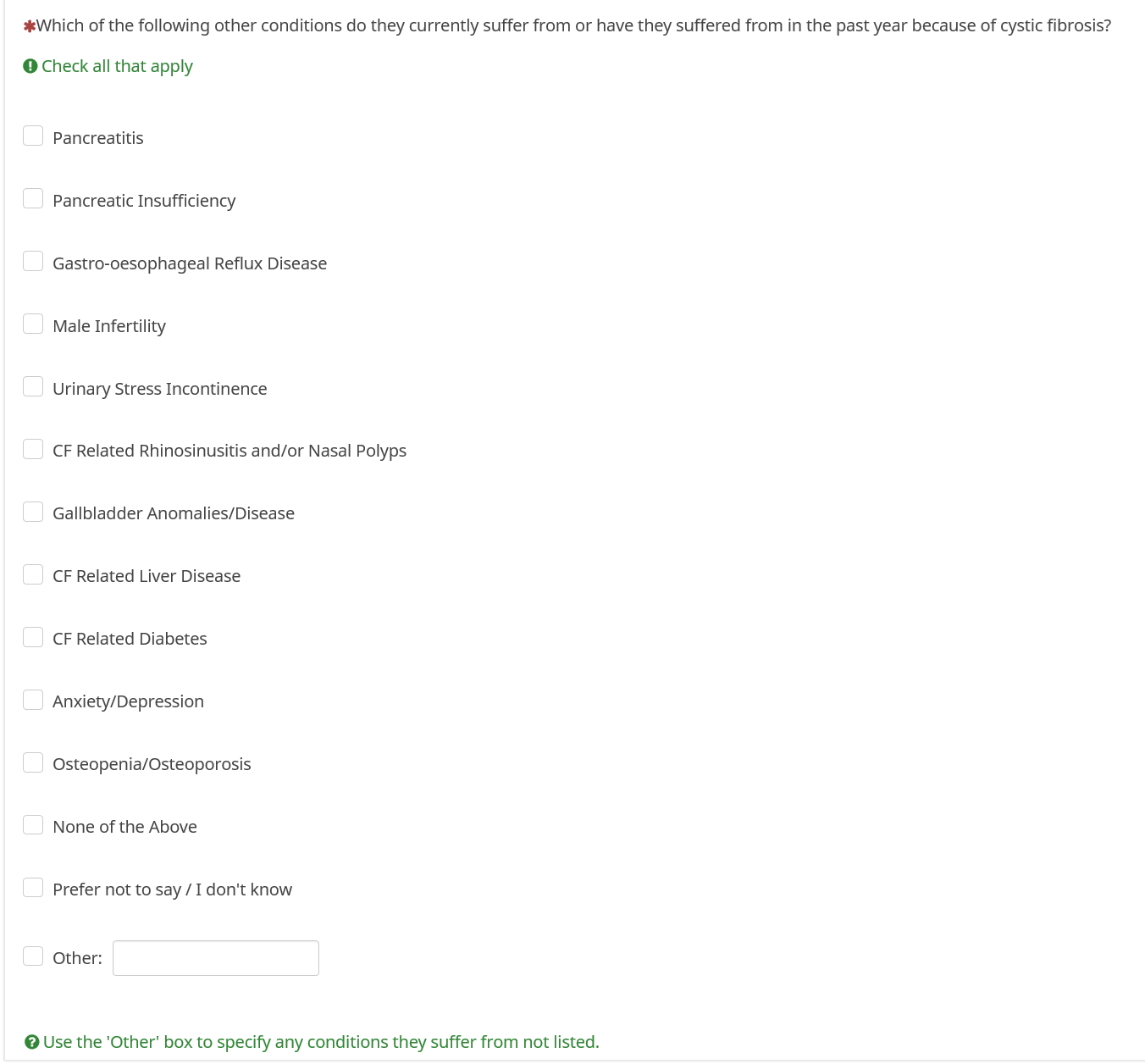

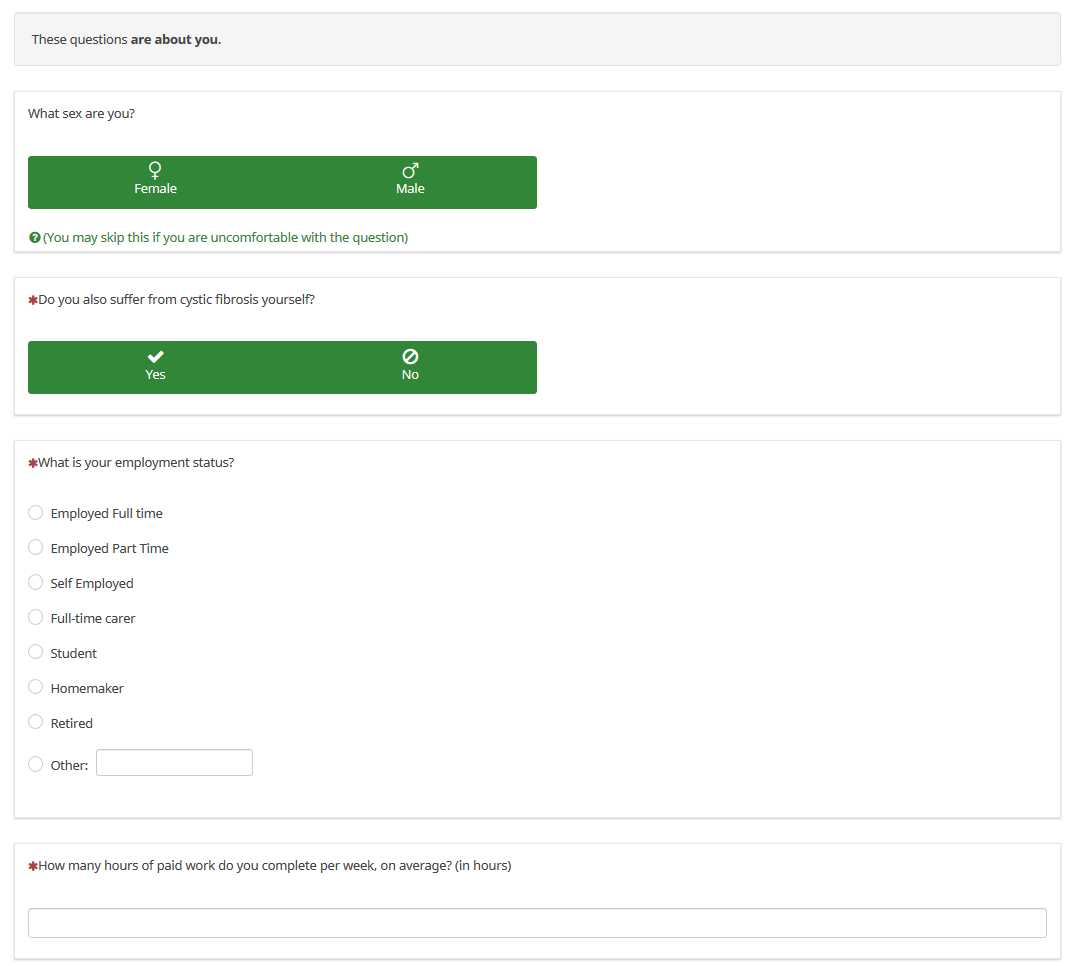


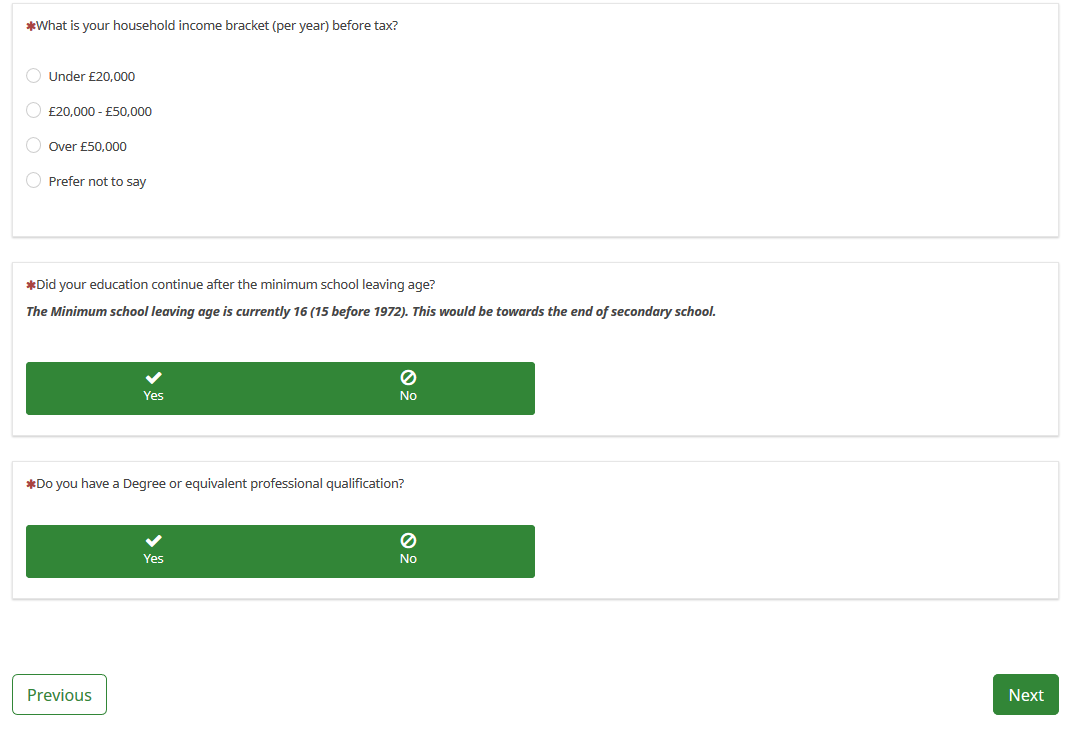

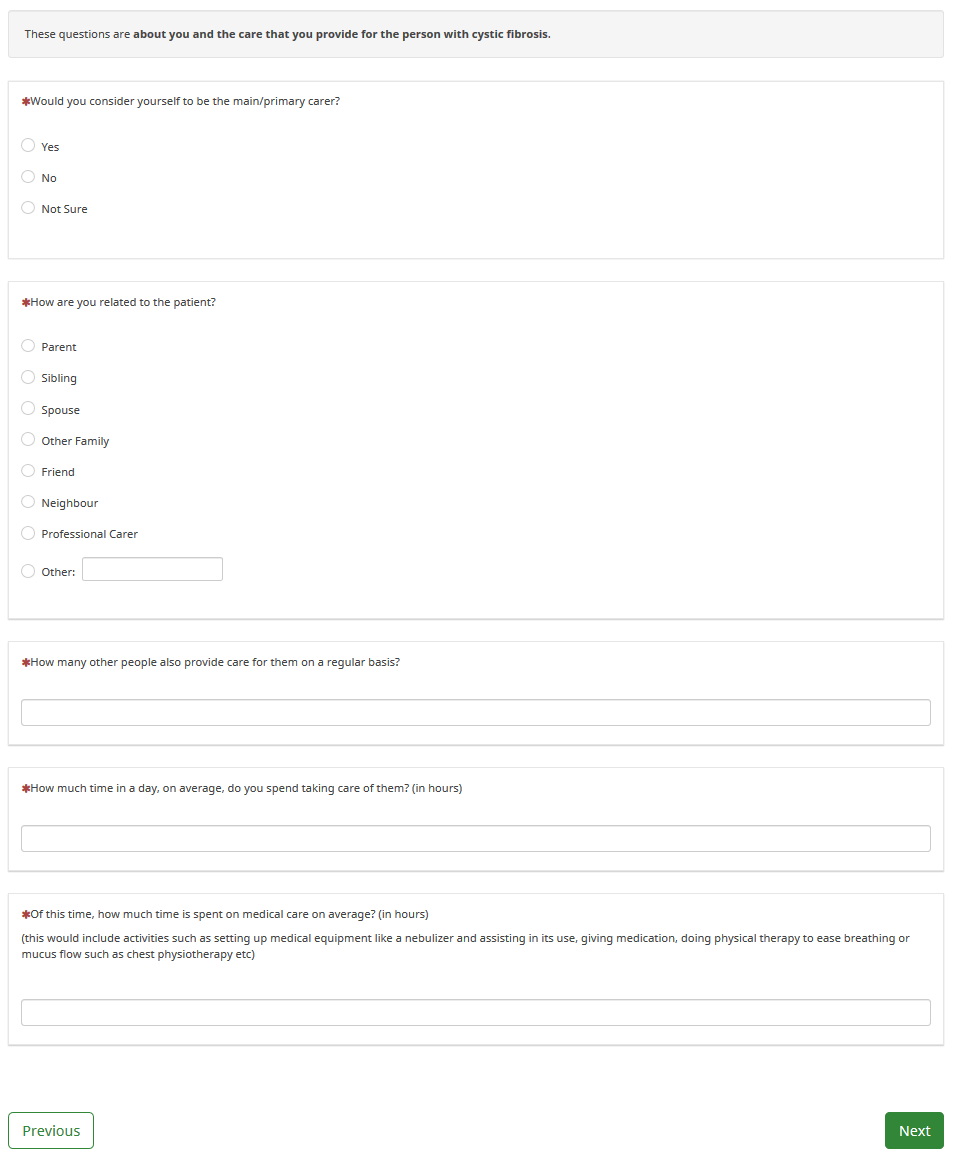


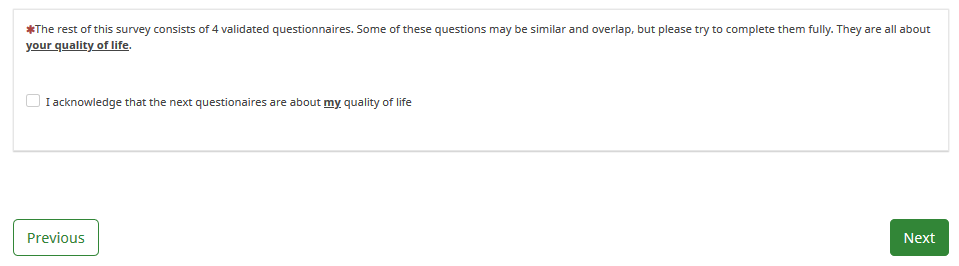

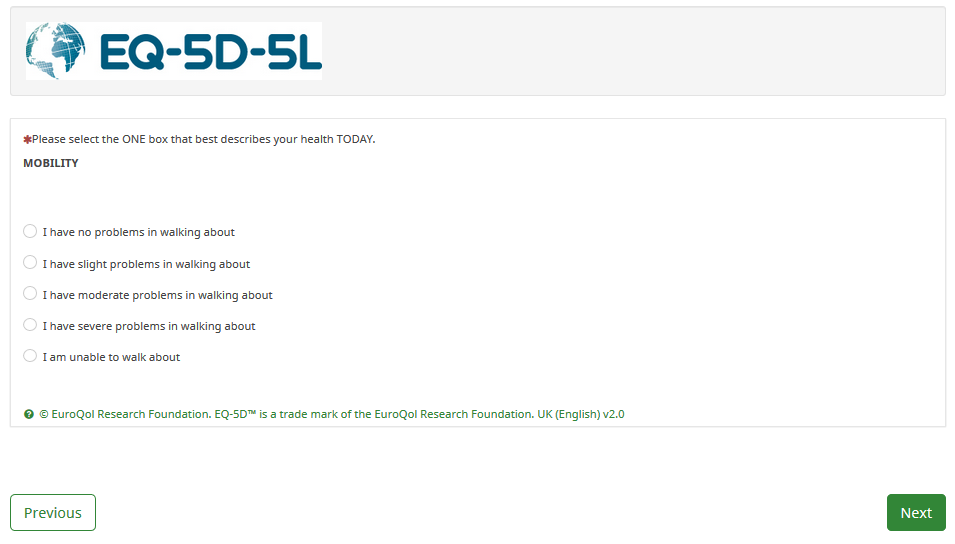

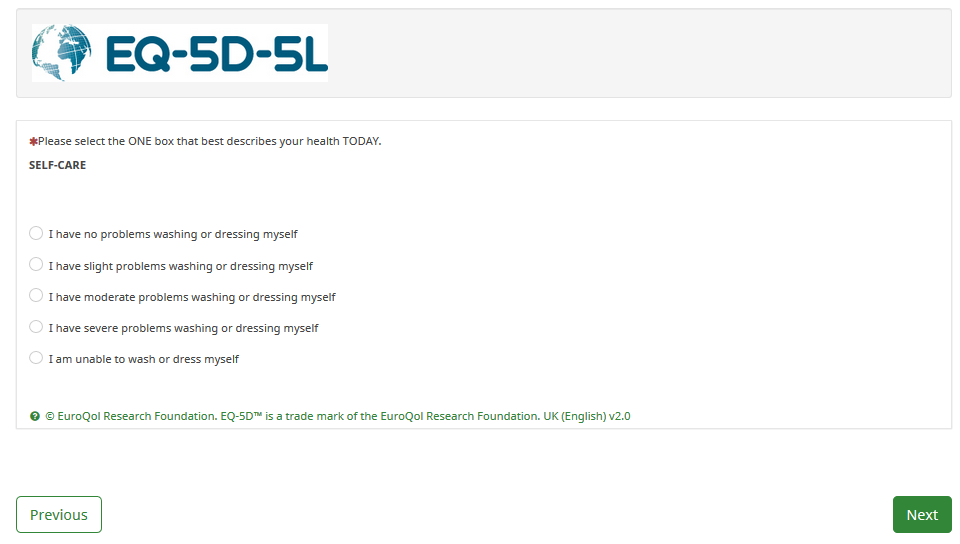


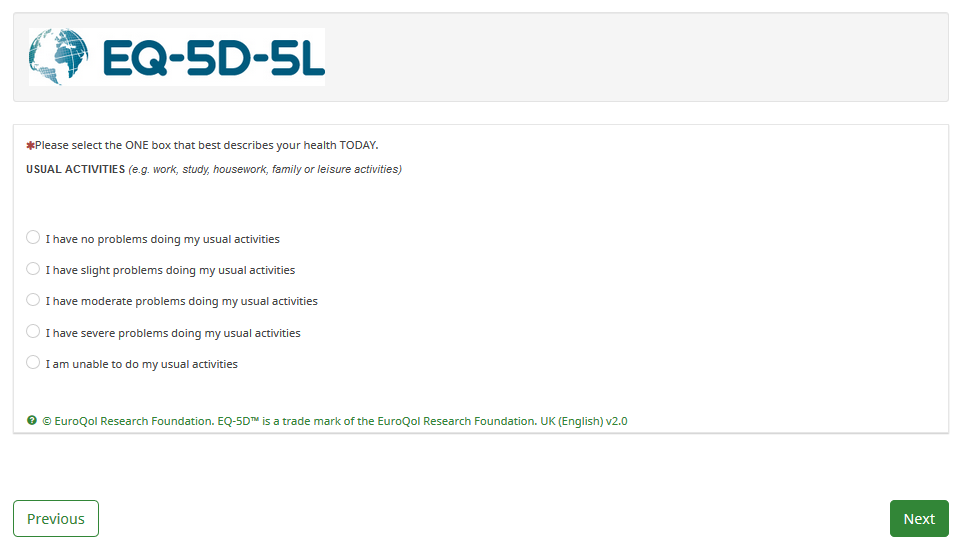

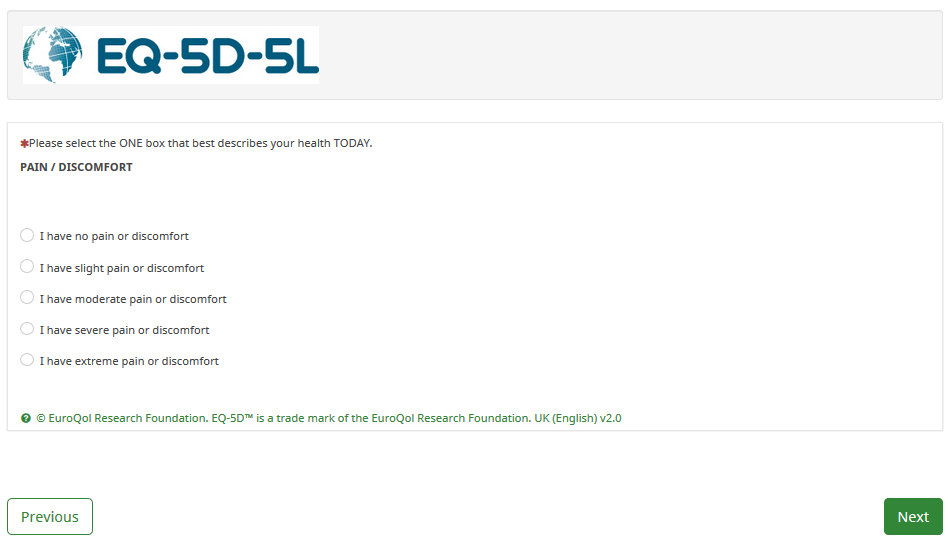

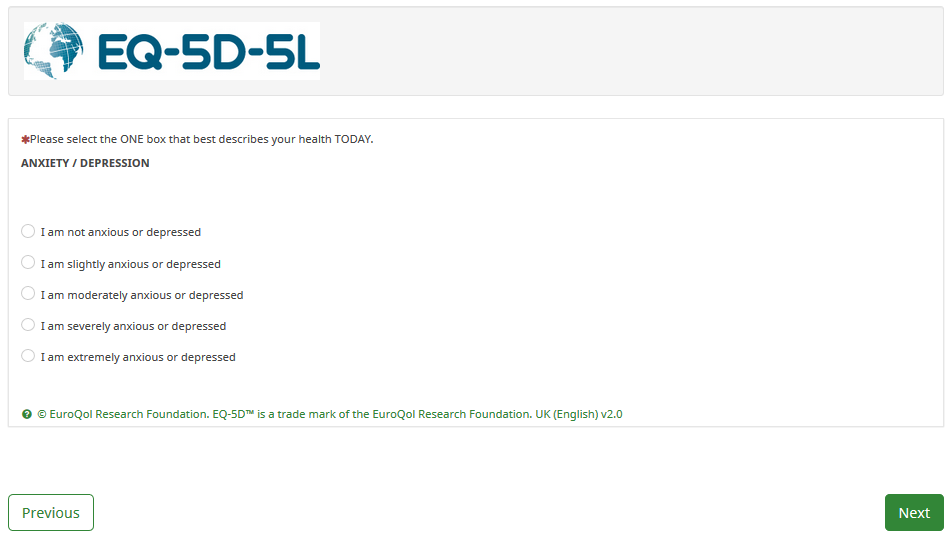


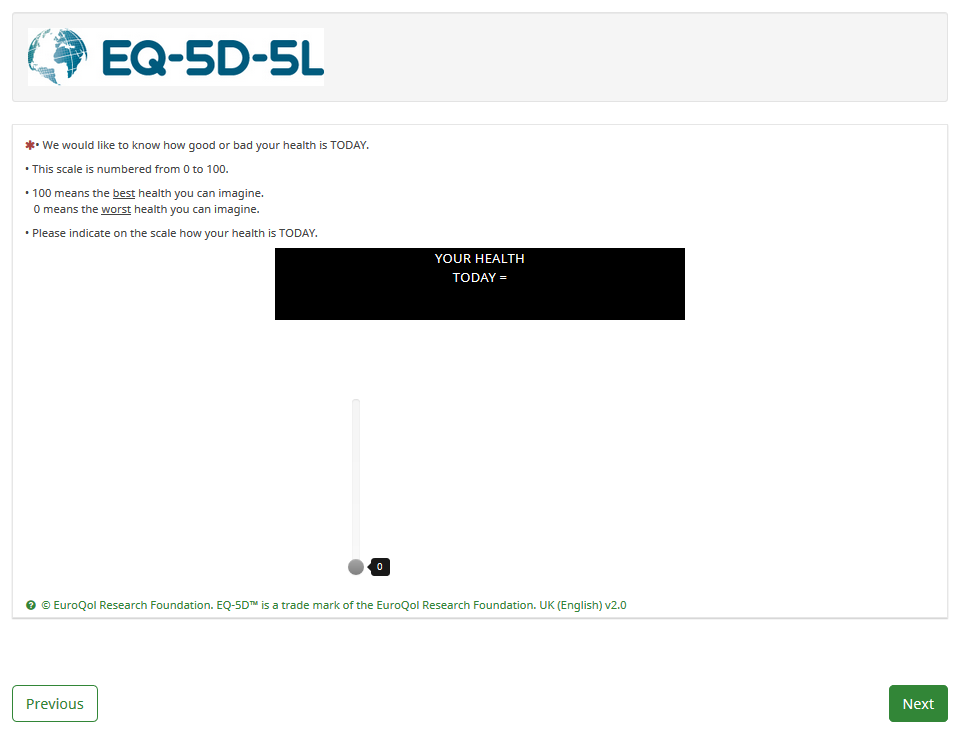

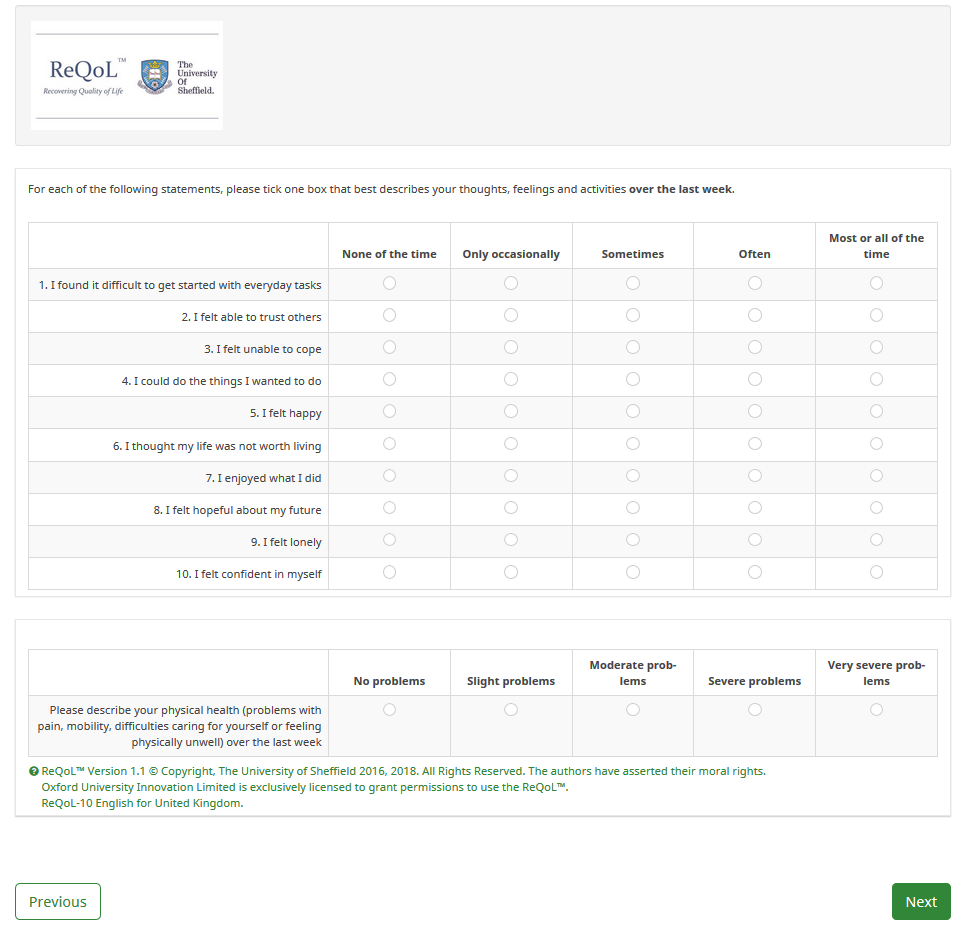


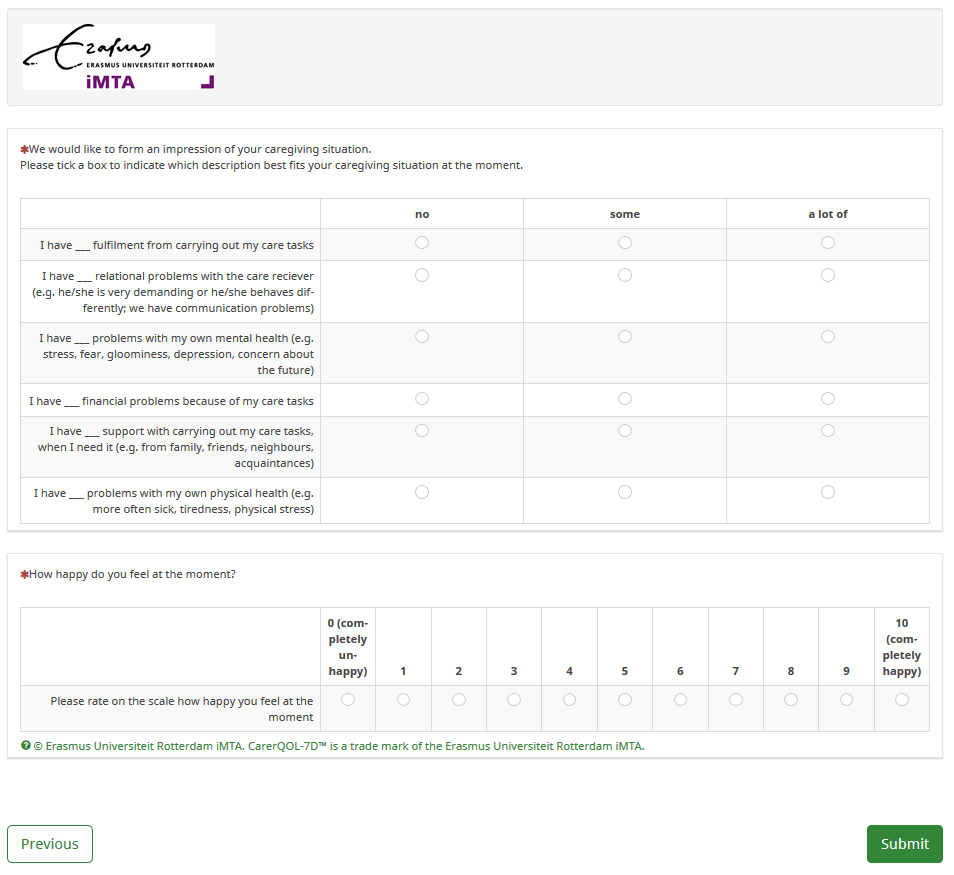

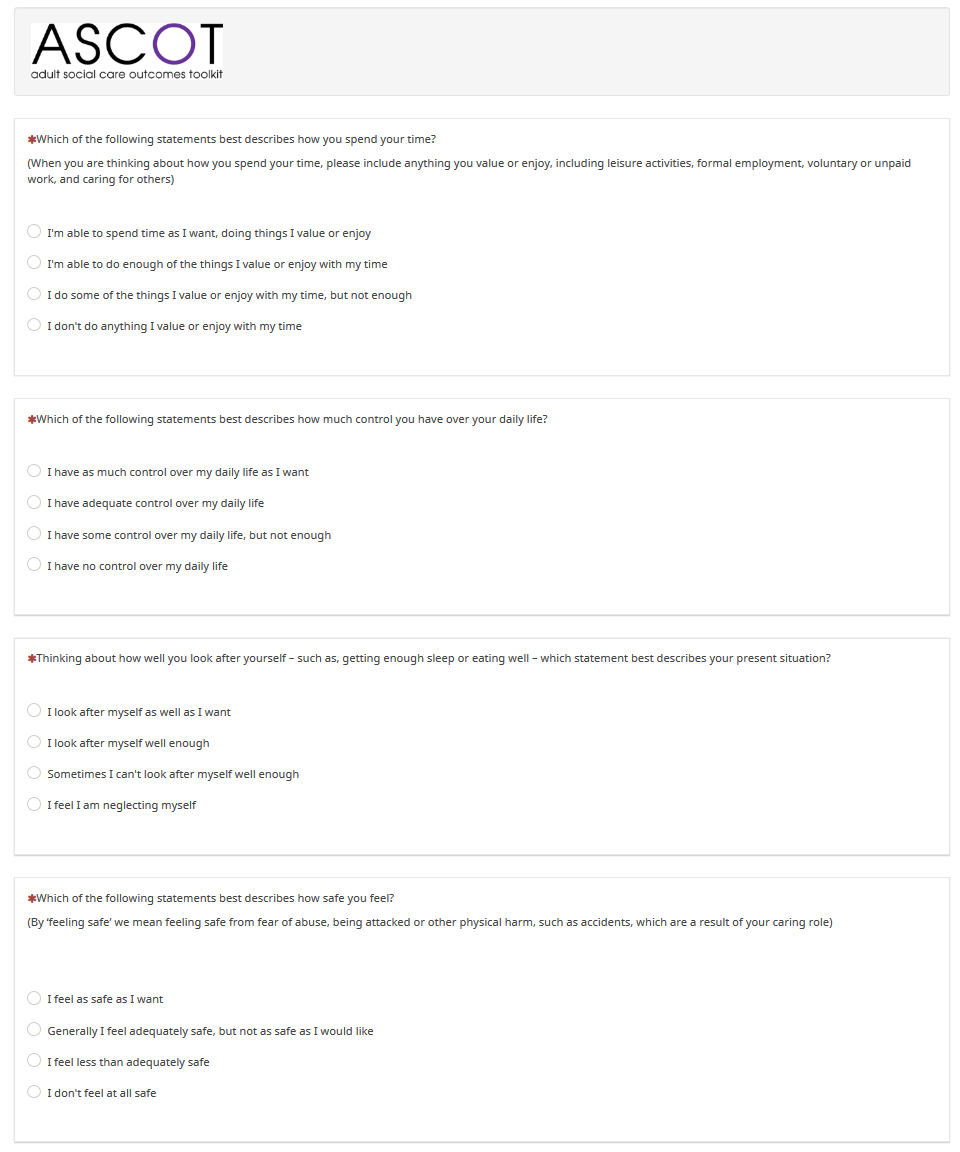

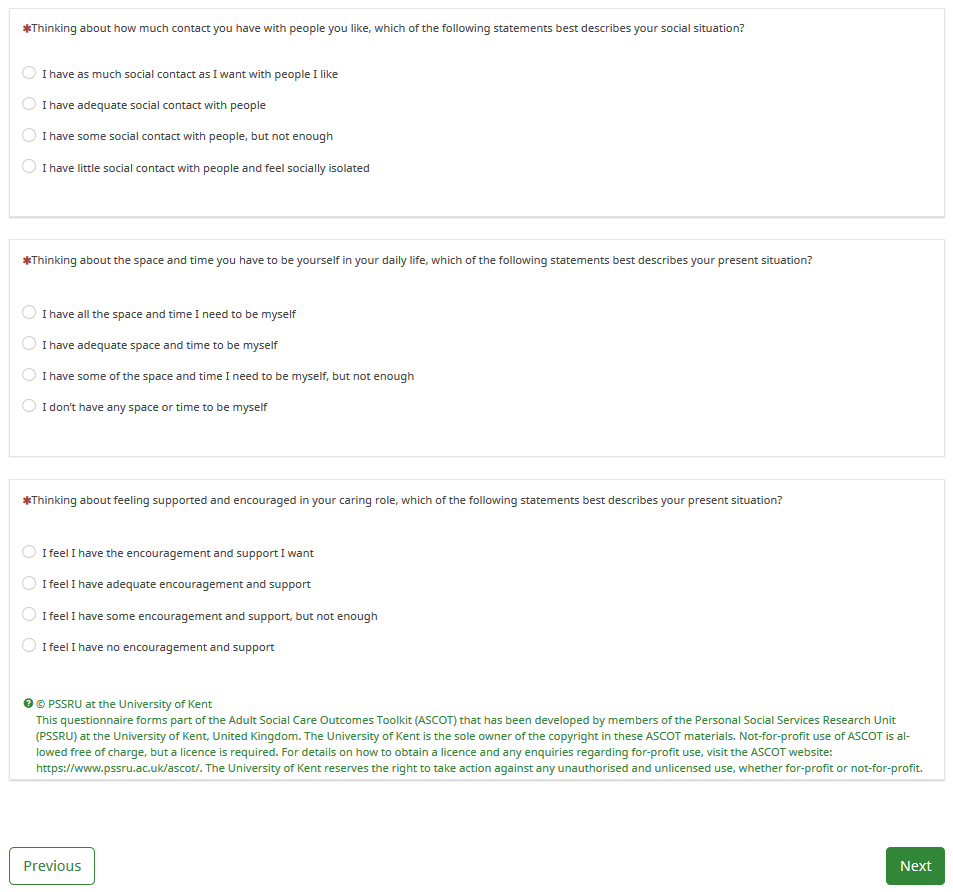

Supplement: Supplementary file 1 — Supplementary file1 (DOCX 1189 KB) [file 11136_2025_4021_MOESM1_ESM.docx]
